# Automated Triage System for Intensive Care Admissions during the COVID-19 Pandemic Using Hybrid XGBoost-AHP Approach

**DOI:** 10.3390/s21196379

**Published:** 2021-09-24

**Authors:** Mohanad A. Deif, Ahmed A. A. Solyman, Mohammed H. Alsharif, Peerapong Uthansakul

**Affiliations:** 1 Department of Bioelectronics, Modern University of Technology and Information (MTI), Cairo 11571, Egypt; Mohand.Deif@eng.mti.edu.eg; 2 Department of Electrical and Electronics Engineering, Istanbul Gelisim University, 34310 Avcılar, Turkey; aaasahmed@gelisim.edu.tr; 3 Department of Electrical Engineering, College of Electronics and Information Engineering, Sejong University, 209 Neungdong-ro, Gwangjin-gu, Seoul 05006, Korea; 4 School of Telecommunication Engineering, Suranaree University of Technology, Nakhon Ratchasima 30000, Thailand

**Keywords:** automated triage, emergency department, intensive care admissions, COVID-19 pandemic, hybrid XGBoost-AHP approach

## Abstract

The sudden increase in patients with severe COVID-19 has obliged doctors to make admissions to intensive care units (ICUs) in health care practices where capacity is exceeded by the demand. To help with difficult triage decisions, we proposed an integration system Xtreme Gradient Boosting (XGBoost) classifier and Analytic Hierarchy Process (AHP) to assist health authorities in identifying patients’ priorities to be admitted into ICUs according to the findings of the biological laboratory investigation for patients with COVID-19. The Xtreme Gradient Boosting (XGBoost) classifier was used to decide whether or not they should admit patients into ICUs, before applying them to an AHP for admissions’ priority ranking for ICUs. The 38 commonly used clinical variables were considered and their contributions were determined by the Shapley’s Additive explanations (SHAP) approach. In this research, five types of classifier algorithms were compared: Support Vector Machine (SVM), Decision Tree (DT), K-Nearest Neighborhood (KNN), Random Forest (RF), and Artificial Neural Network (ANN), to evaluate the XGBoost performance, while the AHP system compared its results with a committee formed from experienced clinicians. The proposed (XGBoost) classifier achieved a high prediction accuracy as it could discriminate between patients with COVID-19 who need ICU admission and those who do not with accuracy, sensitivity, and specificity rates of 97%, 96%, and 96% respectively, while the AHP system results were close to experienced clinicians’ decisions for determining the priority of patients that need to be admitted to the ICU. Eventually, medical sectors can use the suggested framework to classify patients with COVID-19 who require ICU admission and prioritize them based on integrated AHP methodologies.

## 1. Introduction

Severe Acute Respiratory Syndrome CoronaVirus 2 (SARS-CoV-2) has caused the present pandemic of coronavirus disease 2019 (COVID-19) [1]. The first cases of SARS-CoV-2 appeared as an eruption in the Chinese region of Hubei in December 2019 [2].

In the first week of March 2020, over 400,000 cases were confirmed globally, in 130 countries, and by 29 January 2021, the confirmed cases had risen to a little above 100,819,363 million in 250 countries/regions, with over 2,176,159 deaths worldwide [3].

At the beginning of 2021, the number of countries struggling with the COVID-19 pandemic rose to over 250. The number of cases is increasing rapidly in many countries. One of the most important needs in this period in which the severity of the epidemic increased is the number of beds and ventilators (respirators) in ICUs. Intensive care units (ICUs) are critical to improving the survival of patients with serious COVID-19, to supply continuous oxygen help in aided ventilation when needed [4,5], and attention around the clock. ICUs are a valuable asset in areas with a high number of patients with COVID-19 [6].

However, many countries are worried about the lack of health infrastructure in the face of the rapidly increasing number of cases [7]. While governments have applied various protection measures in the process, health units are working to prevent the tsunami caused by a large number of infected individuals to be treated [8]. For instance, Spain and Italy have been hit very hard with tremendous documented cases and deaths [9]. Especially in Italy, critical resources such as protective equipment, ventilators, and even medical staff are becoming deficient. Doctors are being forced to choose to whom care should be prioritized [10].

According to the paper by Emanuel et al. [11], the regular approach of treating people on a “first-come, first-served” basis should not apply during these times. They suggested that prioritizing some indicators related to age, respiratory, and cardiac systems should be a better approach to consider the patients. While the COVID-19 pandemic, which has affected the entire world, caused a noticeable slowdown or even almost complete halt in all businesses and industry, the necessity of overloading the health system and using the health-related resources and health personnel effectively have been revealed.

Since the beginning of the pandemic, a large number of academics have produced significant papers and contributions to the struggle with COVID-19. Although the proposed study focuses on the decisions at the operational level, most of the studies relevant to COVID-19 have concentrated on strategic-level decisions such as spreading models or governments’ policies.

For instance, Giordano et al. [12] proposed a new model that predicts the course of the epidemic to help plan an effective control strategy for Italy. Their discoveries provide policymakers with a tool to assess the consequences of possible strategies, including lockdown and social distancing, as well as testing and contact tracing.

To mitigate the COVID-19 outbreak, Carli et al. [13] proposed an optimal control approach that supports governments in defining the most effective strategies to be adopted during post-lockdown mitigation phases in a multi-region scenario. Then, Pare et al. [14] presented a variety of mathematical models that have been proposed to capture the dynamic behavior of epidemic processes and to estimate the spreading parameters of the virus. For an excellent review of COVID-19 forecasting and SIR models, the reader is referred to Rahimi et al. [15]. Therefore, it is necessary to act immediately and develop systematic methodologies in order to overcome the aforementioned issues, maintain the healthcare system, and fight with the current pandemic by protecting valuable and limited resources and the healthcare personnel.

This research proposes a multi-decision-making procedure (AHP) and XGBoost to aid healthcare professionals in prioritizing patients infected with COVID-19 based on the results of biological laboratory examinations, to provide the desired intensive care facilities, and to manage patients’ health conditions by indoor healthcare providers.

The applied methodology in this paper includes three main phases. In the first part, the XGBoost classifier discriminated patients in a dataset into patients with COVID-19 who need ICU admission and those who do not. Then, the necessary criteria that are considered for ICU admission were determined. It is expected that all the criteria do not have the same priority. For instance, vasopressor need may be more urgent than the arrhythmia problem of a patient for ICU admission. For this reason, the criteria weights were determined using AHP in the second part. Finally, the next question is which patient positive with COVID-19 will use the ICU first in an emergency or limited-resource situation. To answer that question, the criteria weights were applied to rank the patients who need ICU treatment in the last part.

The Analytical Hierarchical Process (AHP) is a multiple-criteria decision-making approach that provides a structured and simple framework for decision-making [16,17]. Medical, information management systems, engineering, financial, geography, business, industry, education, and healthcare sectors have all the used AHP to tackle difficult decision problems [18,19,20]. A set of classification or regression trees is used in XGBoost, which is based on DT ensembles [21]. It predicts a target variable using training data (with multiple features) [22,23].

According to the investigated studies (see Ref. [24]), it can be clearly said that AHP approaches are commonly used in various subsections of healthcare management. In addition, Angelis et al. [25] mentioned that AHP approaches may give a more comprehensive and straightforward approach in healthcare to efficiently capture decision-makers‘ concerns, compare esteem trade-offs, and evoke their esteem inclinations. In expansion, AHP strategies might illuminate the improvement of a choice bolster framework in healthcare, contributing toward more productive, levelheaded, and authentic asset assignment choices.

At the time of writing, there is no research on the integrated system “XGBoost and AHP method” to determine and prioritize the patient status of COVID-19 to refer to health services, but there are other studies that have only prioritized the status without classification steps. This research determined the necessary standards based on knowledgeable human choices, and we studied machine learning methods. On the other hand, other studies on the economic impact of the pandemic on China and the world [26] have used behavioral and social science to support the response to COVID-19, the pandemic [27], the food supply chain during the COVID-19 pandemic [28], etc. Readers can easily find different COVID-19 papers on different topics from different angles.

The authors were motivated to write this paper because they needed to determine the best strategy for accurately separating and prioritizing many patients infected with COVID-19 based on multi-laboratory examination features. If the proposed method is imposed on indoor healthcare providers (such as clinics and hospitals), medical staff are supposed to manage infected patients and distinguish between health conditions for large-scale admissions, as well as ensure treatment equity between treatment structures across affected areas. The paper is organized as follows: a brief introduction and the potential of the proposed solution to the problem are presented in Section 1. The dataset preprocessing and the proposed methodology phases of the prioritization of COVID-19 patients are shown in Section 2. The results are discussed in Section 3, and the conclusion is presented in Section 4.

## 2. Materials and Methods

### 2.1. Materials

The dataset was obtained from the Kaggle online resource [29] to Sirio Libanês, a top-tier hospital in Brazil, which covers the gathered data of prior illnesses, blood sample results, and vital sign data of 1945 patients positive with COVID-19.

There are 54 features: patient’s age demographic information, sexual category, and percentiles. Many patients have pre-existing noncommunicable illnesses (NCDs), such as immunocompromised status and hypertension.

The following blood parameters were examined: aspartate aminotransferase (AST/TGO), international normalized ratio (INR), partial pressure oxygen (PO2) arterial, glucose, heart rate, systolic, base excess venous, hematocrit, oxygen saturation, arterial blood gas test, hemoglobin, bicarbonate venous, bilirubin, O2 saturation arterial, free fatty acid (FFA), PO2 venous, calcium, creatinine, lactate, number of WBCs, neutrophil-to-lymphocyte ratio (NLR), partial pressure carbon dioxide (PCO2) arterial, PCO2 venous, gamma-glutamyl transferase (GGT), pH for arterial, pH for venous, platelets, potassium, venous oxygen saturation, sodium, alanine aminotransferase (ALT/TGP), treponema pallidum particle agglutination assay (TTPA), urea, respiratory rate, temperature, serum albumin base excess arterial, blast, and diastolic blood pressure.

#### Filtering the Dataset and Splitting

There were a lot of missing variables in the Sirio Libanes dataset. The reason for removing entries with missing parameter values is that poor predictive performance was shown in pilot research with the imputation of missing values with mean, median, or regression values. As a result, we excluded entries that had at least one missing value.

This process resulted in 550 sets of patient data entries in the second dataset with no null values. In addition, 264 people in this dataset had severe enough symptoms to be hospitalized in the intensive care unit. The datasets were denoised, and standard scaling techniques were used to accomplish feature scaling, which resulted in the mean value of the data being 0 and the variance value being 1. This can be calculated as

Feature scaling = A feature’s mean value − (Original value/Standard deviation)(1)

All the data were evaluated for statistical analysis after preprocessing. Tenfold cross-validation was used and 80% of the grouped patients’ data were randomly selected for the model training phase and the rest for classifier model validation testing.

To identify the most significant and associative blood parameters, the Student’s t-test was used for continuous variables and Pearson’s correlation among various blood samples counts. The null hypothesis was: the data from both the patient with COVID-19 and healthy population are indistinguishable. Significant blood parameters were chosen based on *p*-value < 0.05.

### 2.2. Methodology

**Figure 1:**
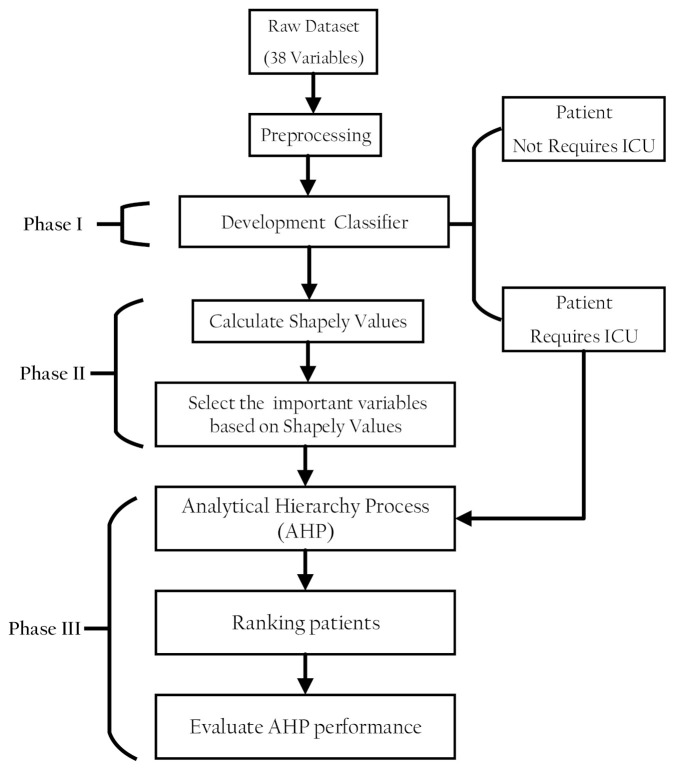
Structure of the research methodology phases.

The proposed methodology comprised three phases. In the first phase, a classification model was developed to discriminate patients in a dataset into patients with COVID-19 who need ICU admission and those who do not. In the second part, the classifier model prediction was interpreted using the SHAP values to select features that have pronounced effects on the classifier decision. In the third part, AHP was used to rank the patients with COVID-19 according to their severity of ICU admission. Figure 1 shows the proposed methodology phases structure.

#### 2.2.1. Phase I Development of a Classification Model

Xtreme gradient achieves the classification model for distinguishing between patients with COVID-19 boosting (XGBoost). A comparison was conducted between the XGBoost classifier and traditional machine learning algorithms [30]: Decision Tree (DT), Random Forest (RF), Support Vector Machine (SVM), K-Nearest Neighborhood (KNN), and Artificial Neural Network (ANN). The Xtreme gradient boosting (XGBoost) schemes and the hyper-parameter setting of other classifiers employed in the experiments were further discussed.

##### Xtreme Gradient Boosting (XGBoost)

**Figure 2:**
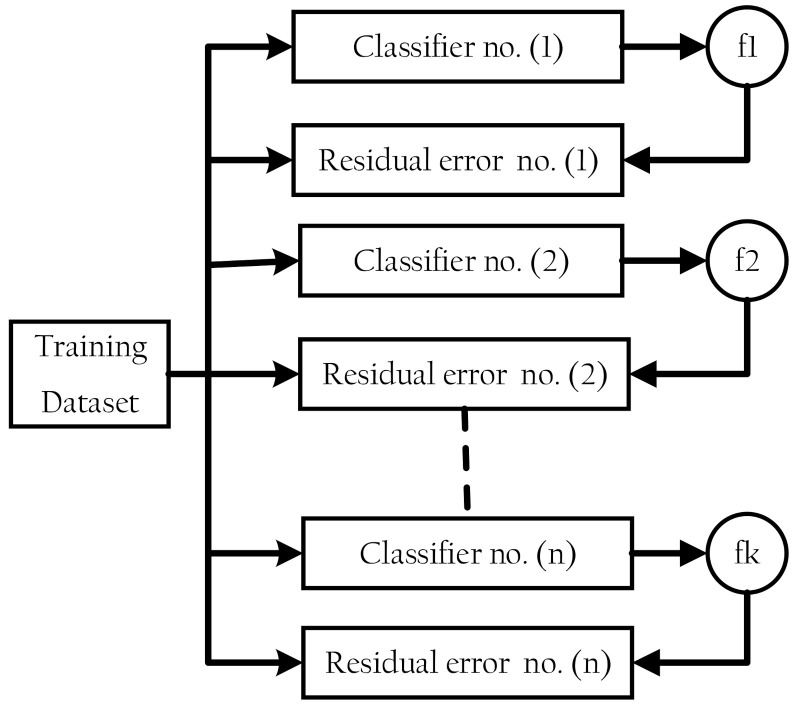
Extreme gradient boosting structure.

XGBoost is a classifier that combines a weak base classifier with a stronger classifier [21,22]. The residual error of a base classifier’s residual is applied in the next classifier to optimize the aim function at each epoch of the training process [31], as shown in Figure 2. DT, SVM, KNN, logistic regression, and other algorithms are among the available base classifiers.

Assuming that the base classifiers are trees with several K, for an input sample $x_{i}$, the classifier output is given by Equation (2):

(2) $${ỳ}_{i}=\sum_{K=1}^{K}f_{k}\left(x_{i}\right),f_{k}$$

where each $f_{k}$ corresponds to a standalone tree with leaf scores. Equation (3) describes the loss function:

(3) $$L\left(f_{t}\right)=\sum \;l\left({ỳ}_{i},y_{i}\right)+\sum \;\Omega \left(f_{t}\right)$$

The first term (l) represents a differentiable loss function, which measures the difference between the predicted output (${ỳ}_{t}$) and the actual output ($y_{i}$). The second term (Ω) represents a regularization part that is used to avoid over-fitting, where Ω and ${ỳ}_{1}$ can be shown as Equations (4) and (5), respectively,

(4) $$\begin{array}{l} {ỳ}_{i}^{\left(t\right)}={ỳ}_{i}^{\left(t-1\right)}+f_{t}\left(x_{i}\right) \end{array}$$

(5) $$\Omega \left(f\right)=\gamma T+\frac{1}{2}∥w{∥}^{2}$$

T denotes the number of leaf nodes and w represents the score on each leaf. As a result, we can conclude that:

(6) $$L\left(f_{i}\right)\approx \sum_{j=1}^{T}\left[(\sum_{kI_{j}}g_{i})w_{j}+\frac{1}{2}(\sum_{kI_{f}}h_{i}+\lambda )w_{j}^{2}\right]+\gamma T$$

where $g_{i}$ and $h_{i}$ are 1st and 2nd order of the loss function. The parameters γ and λ are constants that regulate regularization.

Details of the hyper-parameter setting for the XGBoost model and other traditional classifier algorithms that were used in this work are summarized in Table 1.

The performance of each classifier used in this study was evaluated using sensitivity, specificity, and accuracy tests, which contained true positive (TP), true negative (TN), false negative (FN), and false positive (FP) words. The following formulas were used to calculate these figures:

(7) $$Sensitivity=\frac{TP}{TP+FN}\times 100\%$$

(8) $$Specifity=\frac{TN}{FP+TN}\times 100\%$$

(9) $$Accuracy=\frac{TP+TN}{TP+TN+FP+FN}\times 100\%$$

#### 2.2.2. Phase II: Interpreting the XGBoost Model Prediction Using the SHAP Values

The SHAP method was used to comprehend the significance of the various clinical variables and their effect on the model output, hence pointing out the best indicators for predicting patients infected with COVID-19 disease who need admission to ICUs.

Interpretability can be obtained through summary plots [23,32]. The SHAP summary plot shows how much each predictor contributes, either positively or negatively, to the target outcome variable (whether or not the patient needs admission to an ICU). In addition, it shows the global importance of the features.

Features are organized by the summation of the magnitudes of the SHAP values in all the samples. Assume a classifier model with input variables $x=\left(x_{1},x_{2},\ldots ,x_{p}\right)$, where *p* denotes the number of variables. For an original model $f\left(x\right)$, the explanation model $g\left(x^{\prime }\right)$ with simplified input **x**’ is expressed as:

(10) $$f(x)=g(x^{\prime })=\phi_{0}+\sum_{i=1}^{M}\phi_{i}x_{i}^{\prime }$$

where M denotes the input features number, and $\phi_{0}$ denotes the constant value when all the inputs are missing.

#### 2.2.3. Phase III: Developing the AHP-Decision Support System

The AHP-decision Support System sets subjective weights to the clinical variables that were recommended using SHAP Values. These weights are further used to determine the priority of patients that need to be admitted to ICUs. The following steps represent the procedure of the AHP method.

##### Selection Criteria and Developing the Decision Hierarchy

The decision goal defined for the criteria in AHP is represented as a hierarchy in problem modeling [33]. The decision hierarchy is divided into four levels: Level 1: the decision problem aim (at the top); Level 2: the criteria; Level 3: subcriteria; and Level 4: the set of alternatives. The subcriteria in this study were the clinical variables that were chosen to accord to the SHAPLY value top important features. After that, this criterion was clustered into groups to finally achieve the aim of this study, which was to determine the priority of patients that should be admitted to ICUs. Alternatives here mean patients to be ranked according to their various clinical variables.

##### Construction of Pairwise Comparison Matrix

After constructing the decision hierarchy, a set of pair-wise comparison matrices established weights for each level of the hierarchy. A judgment matrix was constructed as follows:

(11) $$A=\left(\begin{array}{ccccc} I_{11} & I_{12} & \ldots & \ldots & I_{1n}\\ I_{21} & I_{22} & \ldots & \ldots & I_{2n}\\ \vdots & \vdots & \vdots & \ddots & \vdots \\ I_{n1} & I_{n2} & \ldots & \ldots & I_{nn} \end{array}\right)\;\mathrm{where}\;\left\{\begin{array}{l} x_{ii}=1\\ x_{ji}=\frac{1}{I_{ij}} \end{array}\right.$$

Elements $I_{11}$ represent the relative importance of each criterion. The relative importance is measured according to pair-wise comparison scales that were suggested by Saaty [34,35] and are shown in Table 2. Relative scales reflect the level of relative importance as equal, moderate, strong, very strong, and extreme by 1, 3, 5, 7, and 9, respectively. These nine points were used to show each expert’s judgments for each comparison. Experts should critically set these relative scales based on their experience and knowledge. The details of the decision-making team (expert’s judgments) are discussed in Section 2.2.4.

##### Construction of the Normalized DM

After constructing the pair-wise comparison matrix, the next step is the normalization to form the matrix elements on a common scale. Every element of matrix A is normalized by dividing each element in a column by the sum of the elements in the same column to create a normalized pairwise comparison matrix $A_{\mathrm{norm}},\;\mathrm{where}\;A_{\mathrm{norm}}$ is the normalized matrix of $A\left(1\right)$, and $A\left(I_{ij}\right)$ is given by Equation (12). $A_{\mathrm{norm}}$ is described as follows:

(12) $$a_{ij}=\frac{I_{ij}}{\sum_{i=1}^{n}I_{ij}},$$

(13) $$A_{\mathrm{norm}\;}=\left(\begin{array}{ccccc} a_{11} & a_{12} & \ldots & \ldots & a_{1n}\\ a_{21} & a_{22} & \ldots & \ldots & a_{2n}\\ \vdots & \vdots & \vdots & \ddots & \vdots \\ a_{n1} & a_{n2} & \ldots & \ldots & a_{nn} \end{array}\right)$$

##### Calculation of All Priority Values (Eigenvector)

The AHP pair-wise comparison employs mathematical procedures to transform the expert’s judgments into weights for each criterion. Equation (14) can calculate the weights of decision factor i.

(14) $$w_{i}=\frac{\sum_{j=1}^{n}a_{ij}}{n}$$

where n is the number of the compared elements. The AHP measurement steps should produce weights based on the evaluator’s preferences.

##### Calculation of the Consistency Ratio (CR)

CR is calculated in Equation (15):

(15) $$\mathrm{CR}=\frac{\mathrm{CI}}{\mathrm{RI}}$$

The degree of inconsistency is measured by the consistency ratio. The related measure of a pair-wise comparison matrix’s degree of inconsistency is RI. The consistency index (CI) is computed by Equation (16):

(16) $$\mathrm{CI}=\frac{\lambda max-n}{n-1}$$

The random index (RI) is computed by Equation (17):

(17) $$\mathrm{RI}=\frac{1.98\left(n-1\right)}{n}\cdot \mathrm{CI}$$

When the CR of the judgment matrix P is less than 0.1, P is considered having acceptable consistency. Otherwise, the elements in P must be adjusted to achieve satisfactory consistency.

##### Final Scores for the Alternatives (Overall Score of Each Patient’s Condition)

In this step, the weight for each criterion was used to obtain scores for each alternative (patient). This score shows the severity of the patient’s condition:

(18) $$\mathrm{Final}\;\mathrm{scores}\;\mathrm{for}\;\mathrm{the}\;\mathrm{alternatives}\;\left(Q\right)=\sum w_{i}\cdot A_{V}$$

##### Ranking Alternatives (Final Decision)

The set of alternatives (patients) can now be sorted by sorting the value Q in ascending order. Each patient is given the highest priority depending on their highest value.

#### 2.2.4. Evaluation of Proposed Methodology Decision

To evaluate the proposed methodology for prioritizing patients to be admitted into ICUs, the results of the AHP had to be compared with the decision-making team (expert’s judgments). Three decision-making teams were constructed from three different national hospitals in Egypt (Ain Shams University Hospital (El Demerdash), Cairo University Hospital (Kasralainy Hospital), and Asyout University Hospital). Each team comprised three specialized physicians (one specialized in internal medicine and two specialized in critical care units). All physicians had over 10 years of experience.

All various clinical variables of the patients with COVID-19 who were ranked with our proposed system in the experimental part were also given to the decision-making team to take their decisions in prioritizing the patients.

#### 2.2.5. Implementation

Phases I and II were implemented using Jupyter Notebook version 6.0.0 along with Python version 3.4.0, while Phase III was employed using Matlab. Both software ran on a PC with an Intel Core i5 processor and 4 GB of RAM, as well as Windows 10 Professional 64 bit as the operating system.

## 3. Experimental and Results

The experimental procedure comprised three phases. In the first phase, a classification of the datasets was performed by using the XGBoost classifier based on various clinical variables for patients that needed to be admitted to ICUs and those who did not. The performance of the XGBoost classifier was then compared with other classifier algorithms.

The second phase showed the important features that were selected according to their effect on the XGBoost classifier decision-making. In the third phase, five patients were randomly selected from the dataset, and the AHP model was then employed to determine the priority of these patients to be admitted to ICUs based on selected important features that were recommended from the previous phase. In the last step, the AHP model decision was compared with the decision of the decision-making team. In the classification phase, 80% of the 550 various clinical variables of the patients with confirmed COVID-19 were employed for training and the rest for testing.

**Figure 3:**
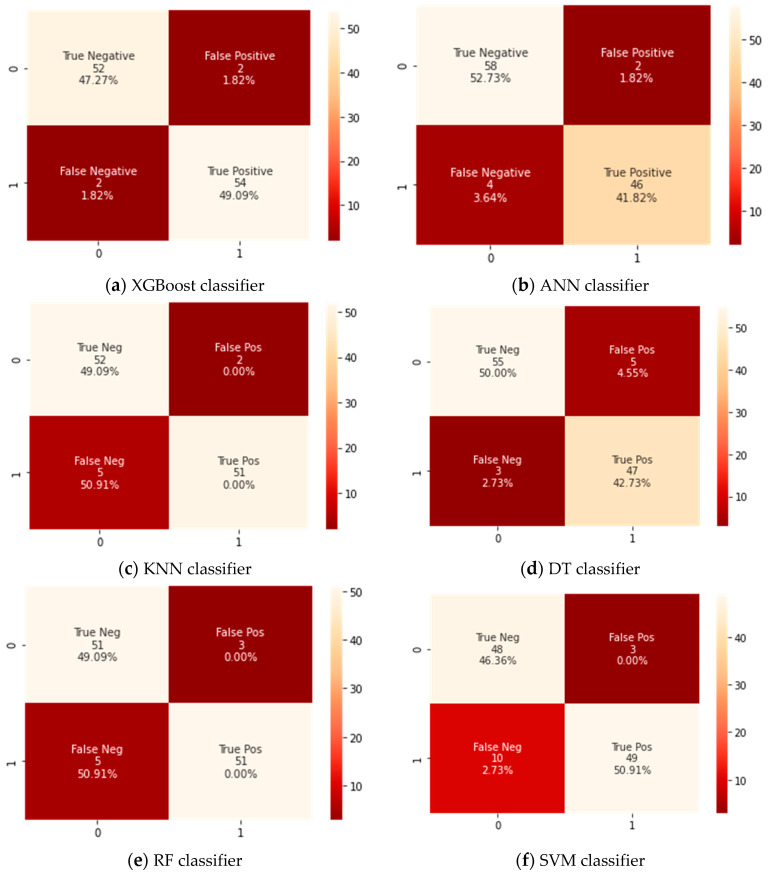
Confusion matrix for all classifiers.

A confusion matrix for the testing dataset that has 110 cases was developed for the Xtreme gradient boosting (XGBoost) classifier and the counterpart classifiers, as shown in Figure 3. A confusion matrix is a technique for summarizing a classification algorithm’s performance. When you have an unbalanced amount of observations in each class or over two classes in your dataset, classification accuracy alone can be misleading. From a confusion matrix, accuracy, sensitivity, and specificity rates were computed and are shown in Table 3.

The findings revealed that the XGBoost model could classify between patients that need to be admitted to ICUs or those who do not by an achieved accuracy of 97%. It was also noticeable that the XGBoost classifier attained a significantly higher accuracy than the corresponding counterpart classifiers did. This is because the confusion matrix as shown in Figure 3 revealed that the tested XGBoost classifier could correctly identify 54 cases having severe symptoms who require admission into ICUs (TP) and 52 cases as patients who do not require to admission to ICUs (TN). Therefore, the XGBoost classifier had achieved a higher accuracy because of its high ability of classification and hence provided a useful and efficient diagnosis of COVID-19 cases that need ICUs using various common clinical variables test data. In addition, the XGBoost classifier had achieved the highest value for the sensitivity of 96% because it had two positively tested cases that were wrongly identified as negatively tested cases (FP) and three negatively tested cases that were wrongly identified as positively tested (FN).

On the other hand, the difference between the specificity and sensitivity values for ANN, KNN, and SVM classifiers was very high, so these classifiers were biased into a certain class. It was also noticed that the specificity rates were higher than sensitivity rates, which means these classifiers were biased to distinguish the cases that do not need admission to ICUs.

DT and RF were almost of the same performance, but their results remained unsatisfactory compared to the XGBoost classifier. The SVM classifier showed the lowest performance and therefore lesser ability of discrimination between cases, even if the settings for the classifier were altered.

**Figure 4:**
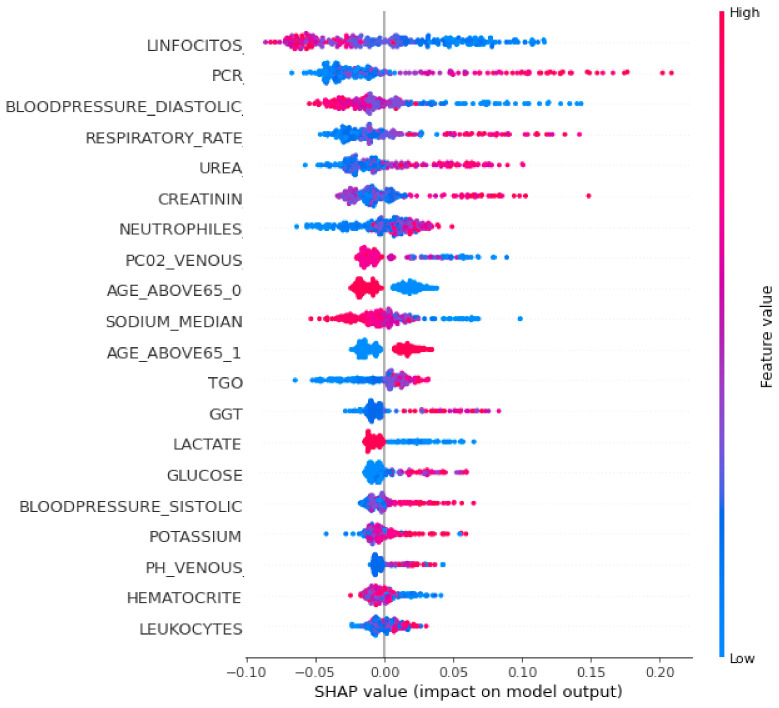
SHAP summary plot of the XGBoost classifier.

To interpret the XGBoost classifier model and to show the relative importance of each feature and its effect on the predicting ability, a SHAP summary plot was performed and is shown in Figure 4. Each point in the SHAP summary plot represents a row of the dataset. It can show the positive or negative relationships for each variable with the target.

Features are sorted in descending order according to their importance. The horizontal location in the SHAP summary plot shows whether the effect of that value is associated with a higher or lower prediction. The x-axis points show the effect of the feature on the estimation of a specific patient. Color refers to either high (red) or low (blue) relative variables. Positive SHAP values show that the model predicted patients with confirmed COVID-19 that need ICUs, while a negative SHAP value shows patients with confirmed COVID-19 who do not need ICUs. SHAP values farther away from zero mean a bigger impact for a certain feature.

It was noticed from Figure 4 that the topmost important clinical variables that had a significant effect on the XGBoost model’s prediction were the lymphocytes, PCR, diastolic blood pressure, respiratory rate, urea concentration, creatinine, neutrophils, P02 venous blood gas, age above 65, sodium, TGO, GGT, glucose, and lactate.

It was observed from Figure 4 that patients predicted by the model who urgently need ICU admission had high values in some features such as the respiratory rate, PCR, urea concentration, creatinine, age, blood pressure, and lymphocytes, and low values in other features such as oxygen saturation, lymphocytes, sodium, hematocrit, and lactate.

We then employed the AHP model to weight each clinical variable that was recommended from the SHAP summary plot. The results of the AHP method were presented after performing all the steps illustrated in Section 2.2.3.

**Figure 5:**
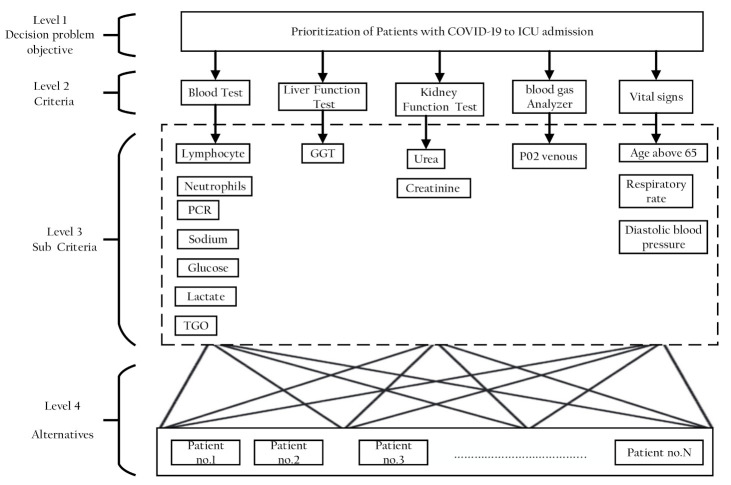
Hierarchy of AHP for clinical variables criteria.

In the first stage of the AHP method, a four-level analytic hierarchical tree was constructed and is shown in Figure 5. The first level was the goal of this study, which is to determine the prioritization of patients with COVID-19 to ICU admission. The second level represents the five key criteria: blood test, liver function test, kidney function test, blood gas analyzer, and vital signs. The third level then shows the detailed composition of the five major criteria into 14 subcriteria: blood test is divided into linfocitos, neutrophils, PCR, sodium, glucose, lactate, and TGO. Kidney function test is divided into urea and creatinine, vital signs is divided into age above 65, respiratory rate, diastolic blood pressure, and liver function, and blood gas analyzer remains as a single criterion GGT and P02 venous, respectively. Afterward, the last level of the decision hierarchy comprises the five patients (alternatives) that need to be ranked to determine the prioritization of patients with COVID-19 to ICU admission based on the selected criteria. The five patients were selected randomly from datasets.

After constructing the decision hierarchy, a set of pair-wise comparison matrices for levels 2 and 3 of the analytic hierarchical tree were created. The pair-wise comparison judgments in this study were obtained through a conversation with the decision-making team. For each of these matrices, pair-wise comparisons were performed between each of the matrix’s two members, using the relative importance scale proposed by Saaty [35].

After constructing the pair-wise comparison matrix, the next step was the normalization to form the matrix elements on a common scale. Then, the computation of criteria weights or vectors of priorities in the matrix was accomplished by applying terms of matrix algebra. After calculating the weights of each criterion in level 2 and subcriteria in level 3, the results were rearranged in descending order of priority. Table 4, Table 5, Table 6 and Table 7 show the weights of the judgment matrix and all priority values (eigenvector) for hierarchy elements in level 2 and 3.

From Table 4, the ranking list of critical criteria showed that the weight of blood tests of 46% occupied the top-most ranking in the list, followed by liver function test (26%), kidney function test, and blood gas analyzer having both weights of 11% and vital signs (6%).

It was noticed from Table 5, Table 6 and Table 7 that the top subcriteria having the highest weights in all lists were the lymphocytes test, urea, and age above 65, while TGO, creatinine, and diastolic blood pressure achieved the lowest weights in all lists. The subcriteria GGT and PO2 venous had the same weights for criteria Level 2 (liver function test and blood gas analyzer).

It was also observed that the CR values shown in Table 4, Table 5, Table 6 and Table 7 were all less than 0.1, which accepted and proved that the expert’s inputs were consistent. After evaluation of the weights for each criterion, the overall score for five patients was computed and is shown in Table 8.

As illustrated in Table 8, the proposed AHP model ranked patient C as the first patient (priority for admission to an ICU) with the highest overall score of 2.116 (28%), patient E as the second in order with an overall score of 1.731 (23%), patient B as the third with an overall score of 1.508 (20%), patient A as the fourth with an overall score of 1.206 (16%), and patient D to be the fifth in order (least priority for admission to an ICU) with a lowest overall score of 0.869 (12%).

To validate the output of this AHP model, the results obtained from the proposed system were compared with the evaluation of the decision-making team for the same five patients that were ranked from the AHP model.

**Figure 6:**
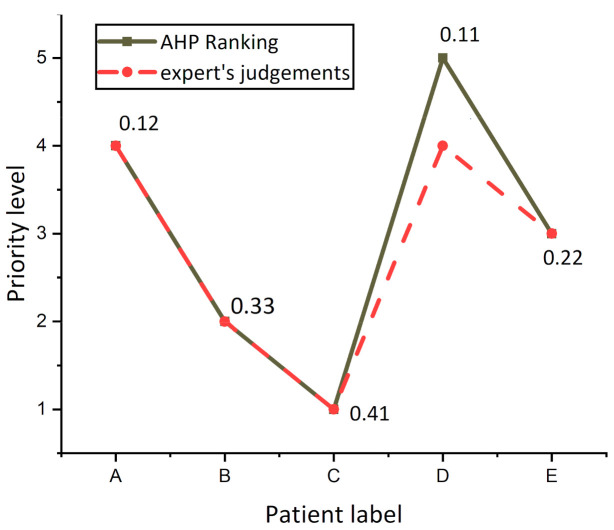
Differences in the patient’s prioritization for the AHP model and decision-making team.

Figure 6 presents the differences between AHP prioritization results (solid line labeled with an overall score of patients) and experts ranking (dash line), and it was observed that the experts ranked patients A, B, C, and E as the same risk level, while experts had varying judgments concerning patient D, because experts evaluated patients A and D as having the same priority level 4. By reference to the value of AHP overall Score of patients, a tiny difference between patients A (0.12) and patient D (0.11) was found, revealing that the evaluation of both the AHP system and experts was the same for patient D.

**Figure 7:**
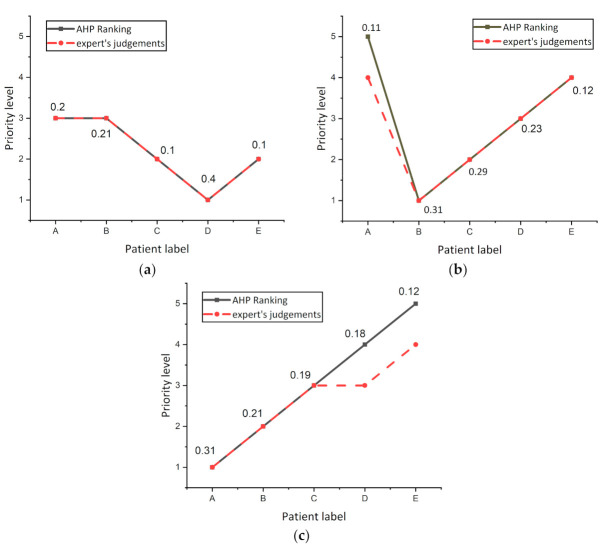
Investigate the variance in AHP and expert’s decision ranking (**a**) Experiment number 1. (**b**) Experiment number 2. (**c**) Experiment number 3.

The experiment was repeated three times on other randomized patients to investigate the variance in AHP and expert’s decision ranking and are shown in Figure 7. It was noticed from the curves that the AHP and expert’s decisions were the same for all patients, while there was a decision variation for patient A in Figure 7b, and patients D and C in Figure 7c. From these results, it was concluded that slight differences between patients in the overall score resulting from the AHP regimen do not show actual differences in the level of risk for this patient. The results showed that when the difference between the total score values of two patients in the AHP is less than 0.01, both patients are at the same risk level.

## 4. Conclusions

Pandemics exert a severe burden on healthcare systems by causing abrupt increases in hospital admissions. The current COVID-19 outbreak has put the entire world at risk, with countries such as Italy, Brazil, Spain, the United States, and the United Kingdom being hit worse than others, even though hospitals have implemented systems to prioritize ICU and ventilator admissions when demand exceeds capacity. Clinicians who must decide who receives potentially life-saving care face a considerable psychological cost while making these judgments. As there may be a trade-off between saving one patient’s life and saving another’s, the ability to construct automated triaging admissions to assess the impact of the epidemic on ICU bed capacity utilization is a vital component of effective outbreak management. This study has made two contributions to the problem of managing ICU capacity during the COVID-19 pandemic peak. The first contribution is the development of a classifier model for predicting patients who needed ICU admissions based on various clinical variables. The second contribution concerns the assignment of the various important clinical variables for patients by SHAPLY value and weighing via the Analytic Hierarchy Process (AHP) method that ranked patient’s priority to ICU admission based on level of risk. The results’ contributions of this study showed that the Xtreme gradient boosting (XGBoost) classifier achieved better performance as compared to the other counterpart frequently used classification models. Moreover, the ranking decision of the AHP model for patients that needed ICUs was very close to the ranking of the decision-making team. We expect that this research can help practitioners and policymakers better allocate resources and enhance patient outcomes for patients with COVID-19.

## Figures and Tables

**Table 1:** The hyper-parameter settings for different classifier algorithms.

Classifier Type	Hyper-Parameter	Optional
XGBoost	Max depth	6
	learning rate	0.1
	The optimum number of estimators	1000
DT	Random state	42
	Measures of impurity by	Gini Index
	Minimum sample split	2
RF	Minimum sample split	2
	Number of estimators	100
SVM	Kernel type	polynomial
	Cache size	200
	Degree of the polynomial kernel	3rd degree
KNN	Distance metrics	Minkowski
	Weights	uniform
	Number of neighbors	3 (k = 3)
ANN	Activation function for all the hidden layers	ReLU (Rectified Linear Units)
	The activation function for the output layer is	Softmax
	The number of epochs	1000 epochs
	Optimization method	Stochastic gradient descent (SGD)
	Learning rate	0.0001

**Table 2:** Nine scales of pairwise comparisons.

Intensity of Importance	Definition	Explanation
1	Of the same importance	Two actions contribute equally to the goal.
3	One has a lower priority than the other.	One activity has a minor advantage over the others gained through experience and judgment.
5	The importance that is essential or strong	One activity is strongly favored over another by experience and judgment.
7	Demonstrated importance	Activity is strongly favored and its dominance is demonstrated in practice
9	Absolute impact	The evidence that supports one action over another is of the highest grade.
2,4,6,8	Intermediate values between the two adjacent judgments	When you need to find a middle ground

**Table 3:** Comparison between XGBoost model and state-of-the-art methods.

Performance Metrics	XGBoost	ANN	KNN	DT	RF	SVM
Accuracy	97%	95%	94%	93%	93%	88%
Sensitivity	96%	92%	91%	94%	91%	83%
Specificity	96%	97%	96%	92%	94%	94%

**Table 4:** Decision matrix and weight calculation for Level 2 criteria.

	Blood Test	Liver Function Test	Kidney Function Test	Blood Gas Analyzer	Vital Signs	Weight	Weight %
Blood Test	1	3	3	5	5	0.46	46%
Liver Function Test	0.3	1	3	5	3	0.26	26%
Kidney Function Test	0.3	0.3	1	1	2	0.11	11%
blood gas Analyzer	0.2	0.2	1	1	4	0.11	11%
Vital signs	0.2	0.3	0.5	0.3	1	0.06	6%
C. R. % =	9.05						

**Table 5:** Decision matrix and weights computation for Level 3 subcriteria derived from blood test criteria.

	Lymphocytes	Neutrophils	PCR	Sodium	Glucose	Lactate	TGO	weight	Weight %
Lymphocytes	1	2	3	3	2	1	2	0.23	23%
Neutrophils	0.5	1	3	3	3	3	3	0.25	25%
PCR	0.3	0.3	1	1	2	2	2	0.13	13%
Sodium	0.3	0.3	1	1	4	2	3	0.15	15%
Glucose	0.5	0.3	0.5	0.3	1	2	2	0.09	9%
Lactate	1	0.3	0.5	0.5	0.5	1	1	0.08	8%
TGO	0.5	0.3	0.5	0.3	0.5	1	1	0.07	7%
C. R. % =	9.32								

**Table 6:** Decision matrix and weights computation for Level 3 subcriteria derived from kidney function test criteria.

	Urea	Creatinine	Weight	Weight %
Urea	1	3	0.75	75%
Creatinine	0.3	1	0.25	25%

**Table 7:** Decision matrix and weights computation for Level 3 subcriteria derived from vital signs criteria.

	Age above 65	Respiratory Rate	Diastolic Blood Pressure	Weight	Weight %
Age above 65	1	3	4	0.61	61%
Respiratory rate	0.3	1	3	0.27	27%
Diastolic blood pressure	0.3	0.3	1	0.12	12%
C. R. % =	6.34				

**Table 8:** Overall score of each patient’s condition.

		Weight	Patient A	Patient B	Patient C	Patient D	Patient E
Blood Test	Lymphocytes	0.23	0.309	0.385	0.783	0.483	0.339
	Neutrophils	0.25	0.407	0.538	0.642	0.655	0.179
	PCR	0.13	0.309	0.423	0.642	0.379	0.107
	Sodium	0.15	0.235	0.554	0.642	0.379	0.179
	Glucose	0.09	0.012	0.308	0.547	0.103	0.214
	Lactate	0.08	0.160	0.092	0.509	0.241	0.286
	TGO	0.07	0.136	0.338	0.245	0.379	0.571
Liver Function Test	GGT	0.26	0.358	0.323	0.264	0.379	0.071
Kidney Function Test	Urea	0.75	0.328	0.386	0.632	0.175	0.929
	Creatinine	0.25	0.365	0.434	0.737	0.320	0.429
blood gas Analyzer	P02 venous	0.11	0.402	0.482	0.789	0.031	0.929
Vital signs	Age above 65	0.61	0.440	0.529	0.737	0.155	0.429
	Respiratory rate	0.27	0.477	0.577	0.579	0.031	0.929
	Diastolic blood pressure	0.12	0.514	0.625	0.579	0.113	0.429
Overall Score of patients			1.206	1.508	2.116	0.869	1.731
Overall Score of patients %			16%	20%	28%	12%	23%

## Data Availability

Not applicable.
